# Antiproliferative activities of the second-generation antipsychotic drug sertindole against breast cancers with a potential application for treatment of breast-to-brain metastases

**DOI:** 10.1038/s41598-018-33740-0

**Published:** 2018-10-25

**Authors:** Wei Zhang, Cunlong Zhang, Feng Liu, Yu Mao, Wei Xu, Tingting Fan, Qinsheng Sun, Shengnan He, Yuzong Chen, Wei Guo, Ying Tan, Yuyang Jiang

**Affiliations:** 1 State Key Laboratory of Chemical Oncogenomics, the Graduate School at Shenzhen, Tsinghua University, Shenzhen, 518055 P. R. China; 2 Department of Pharmacology and Pharmaceutical Sciences, School of Medicine, Tsinghua University, Beijing, 100084 P. R. China; 3 Shenzhen Technology and Engineering Laboratory for Personalized Cancer Diagnostics and Therapeutics, Shenzhen Kivita Innovative Drug Discovery Institute, Shenzhen, 518055 P. R. China; 4 School of Medicine, Tsinghua University, Beijing, 100084 P. R. China

## Abstract

Epidemiological observations have shown that schizophrenia patients after long-term drug treatment exhibited reduced tumor incidences. The potential anticancer effects of antipsychotic drugs are subsequently demonstrated. These drugs are of great interest as agents against untreatable brain metastases because of their ability to traverse the blood-brain barrier (BBB). Most drugs tested thus far are the first-generation antipsychotics (FGAs). But their clinical application may be limited due to high risks of deaths in elderly patients. There is an urgent need to find additional BBB-traversing anticancer agents with lower risks of deaths. In this work, we investigated antitumor activities of eight second-generation-antipsychotic (SGA) drugs, since they exhibit lower mortality rates than FGAs. We discovered that sertindole showed broad antiproliferative activities against seven cancer types including 29 cell-lines and exhibited potent effects toward breast cancer cell-lines, with half maximal concentration to inhibit proliferation by 50% (IC_50_) as low as 800 nM. We further found that sertindole caused cell death through autophagy-associated apoptosis and its directly-binding inhibition of 5-HT6 involved in this process. In xenotransplant mice, sertindole administration approaching maximal therapeutic dose attenuated breast-tumor growth by 22.7%. Therefore, our study reveals promising anticancer potentials of sertindole against breast cancers, with probable applications for breast-to-brain metastases.

## Introduction

An estimated 170,000 cancer patients with brain metastases (BrM) are diagnosed annually in the United States^1,2^. Specific cancer types are especially inclined to metastasize to brain, such as breast cancer, lung cancer and melanoma^3–5^. However, the inability of most anticancer drugs (including chemo-, targeted and immunotherapeutic drugs) to effectively cross the blood-brain barrier (BBB) has represented a significant challenge for BrM treatment^3–5^.

Antipsychotic drugs are currently being explored as potential anticancer agents against BrM^6–20^. First, epidemiological investigations have demonstrated that schizophrenic patients often exhibit reduced tumor incidences after receiving long-term drug treatment^21–23^. Therefore, the repurposing of some antipsychotic drugs for prevention or therapy of cancers may be of value. Moreover, the additional ability of these drugs to cross the BBB makes them attractive candidates for use against BrM.

Almost all anti-cancer drug candidates identified thus far have been first-generation antipsychotics (FGAs)^6–15^. A number of reports have elucidated that FGAs is associated with a spontaneous death risk, especially in elderly patients^24–28^. In patients older than 65 years of age, receiving therapeutic dosages of FGAs induced a ~14-fold higher risk ratio of death, compared with the observation in patients younger than 44 years of age^26–28^. Furthermore, to achieve tumor inhibition, higher working doses of these FGAs are frequently required^14^; such doses are exponentially higher than maximal therapeutic doses used for treatment of psychosis, and may lead to further increases in deaths. This concern, coupled with the fact that patients older than 65 years of age account for ~60% of annually-diagnosed cancer patients^29^, underscores the challenges that must be overcome before FGAs can be safely used for cancer therapy. Meanwhile, death rates of patients taking second-generation antipsychotics (SGAs) are lower than FGAs^24–28,30^. Indeed, a reduction of ~37–50% in deaths was observed for SGAs vs. FGAs in treatment of the elderly population^26–28^. Therefore, until FGAs can be demonstrated to exhibit significant antitumor activities within safe therapeutic dosage ranges, SGAs may be clinically more advantageous.

So far, several SGA agents, such as clozapine, risperidone and olanzapine, have been reported to show moderate antitumor activity in cell tests *in vitro*, exhibiting IC_50_ values (half maximal concentration to inhibit proliferation by 50%) greater than 20 µM^16–20^. Consequently, these results have justified screening of additional SGAs for anticancer activity against BrM. In this work, we measured the antiproliferative activity of eight SGA agents toward the triple-negative breast cancer (TNBC) cell line SUM159. SUM159 was used as our study model due to the high frequency of metastasis of TNBC cells to brain^2^. In our results, one SGA drug, sertindole exhibited appreciable antiproliferative activity toward SUM159 cells. This result prompted us to measure the effects and selectivity of sertindole toward cell lines of two subtypes of breast cancer, ER^−^PR^−^ and ER^+^PR^+^. Subsequently, we further evaluated the breadth and selectivity of sertindole against a panel of 29 cell lines representing seven cancer types. We also studied the mechanism of sertindole action during the induction of cell death and identified protein(s) that directly bind(s) to sertindole to ultimately trigger the antitumor effects. Finally, we used a TNBC orthotopic model to explore antitumor effects of sertindole *in vivo*.

## Results

### Sertindole inhibits proliferation and migration of breast cancer cells

**Figure 1:**
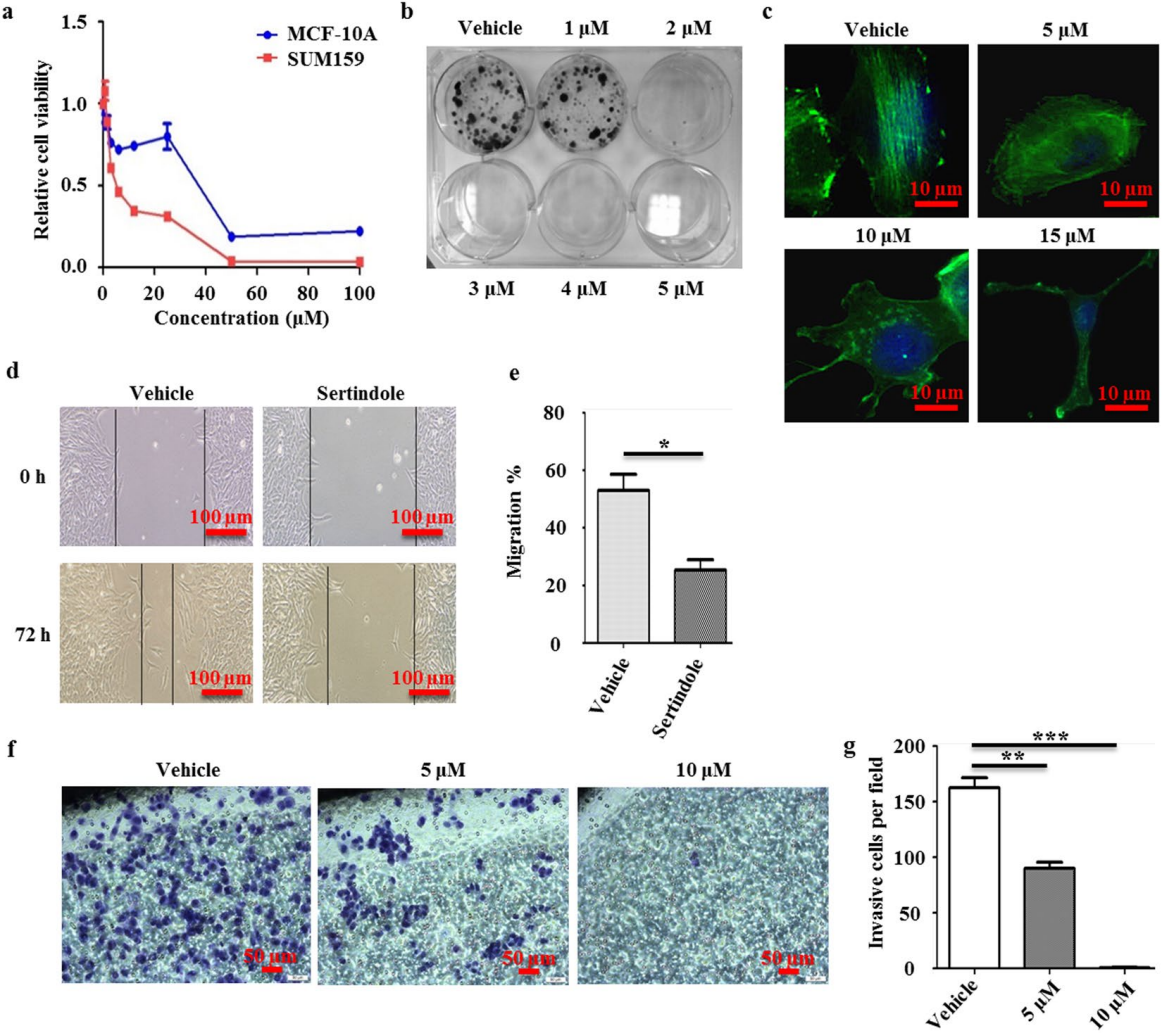
Sertindole attenuates proliferation and migration of breast cancer cells. (**a**) Cell viability curve of SUM159 and MCF-10A cells treated with eight concentrations of sertindole for 48 h. Error bars represented the mean of four ± S.D. (**b**) Colonies of SUM159 cells treated with or without sertindole for 15 days. (**c**) Actin staining of SUM159 cells treated with various concentrations of sertindole for 6 h. Green, phalloidine. Blue, DAPI. Scale bars, 10 µm. (**d**,**e**) Migration of SUM159 cells treated with or without 20 µM sertindole for 72 h. The widths of wounds were measured at 0 h and 72 h after scratching. Width_migration_ = Width_0 h_ − Width_72 h_. (**f**,**g**) Invasion of SUM159 cells in an invasion assay, treated with or without sertindole for 24 h. Stained cells represented the fraction of cells that migrated from the top side of the membrane to the bottom side of the membrane of the insert. Numbers of cells were counted randomly in three fields of views. Error bars represented the mean of triplicates ± S.D. *P < 0.1, **P < 0.05, ***P < 0.01.

First, we measured antiproliferative activities of eight SGA drugs (Supplementary Table S1). With our focus on breast-to-brain metastases, we selected a human TNBC cell line, SUM159, as the study model^2^. We found that three of eight agents displayed cytotoxicity toward these cells. Among them, sertindole showed the best performance (IC_50_ = 9.2 µM), whereas asenapine (IC_50_ = 55.3 µM) was only slightly more effective than positive control clozapine (IC_50_ = 62.8 µM) (Supplementary Table S1). Therefore, sertindole was tested for differential selectivity toward tumor cells vs. non-tumor cells. A toxic effect of sertindole was observed toward MCF-10A cells (an immortalized breast epithelial cell line), with an IC_50_ value of 27.6 µM. However, MCF-10A cells exhibited lower sensitivity toward sertindole than SUM159 cells (Fig. 1a).

Based on its potent antiproliferative activity and selectivity for tumor cells, sertindole was selected for further study. In order to evaluate the minimum sertindole concentration that could attenuate SUM159 cell proliferation, we performed a colony formation assay. Subsequently, colony counts were reduced to 77.5% using 1 µM sertindole, with further reduction to 4.9% observed using 2 µM sertindole (Fig. 1b).

Next, to determine the potential anti-metastatic activity of sertindole towards SUM159 cells, we primarily observed F-actin in stress fibers of cells treated with or without sertindole for 6 h. Stress fibers of cells treated with vehicle displayed straight-lined and well organized. In contrast, in sertindole-treated cells, the stress fibers exhibited disorganized and partially destroyed even at the low concentration of 5 µM. With increased concentrations of sertindole, the cellular cytoplasm was over stretched and the stress fibers were entirely packed together. Finally, the cytoplasm was separated from the cell body (Fig. 1c). Furthermore, we also utilized a wound healing assay to evaluate cell migration. Because this assay requires a cell layer at high confluences (>95%), we had concerns that such growth density might reduce sertindole sensitivity toward the cells^31^, prompting us to perform a pilot study measuring the IC_50_ response of SUM159 cells to sertindole under this condition. Subsequently, the IC_50_ value increased to 30.6 μΜ (Supplementary Fig. S1), prompting the use of 20 μΜ sertindole in the following wound healing assay. As a result, sertindole significantly delayed migration by 52.1% (Fig. 1d,e). Additionally, we measured invasion ability of sertindole-treated SUM159 cells. After 24-hour treatment, we found that 5 μΜ sertindole blocked around 50% cells traversing the membranes, and as the concentration increased to 10 μΜ, almost all the cells lost traversing ability (Fig. 1f,g).

### Sertindole induces autophagy-associated apoptosis

**Figure 2:**
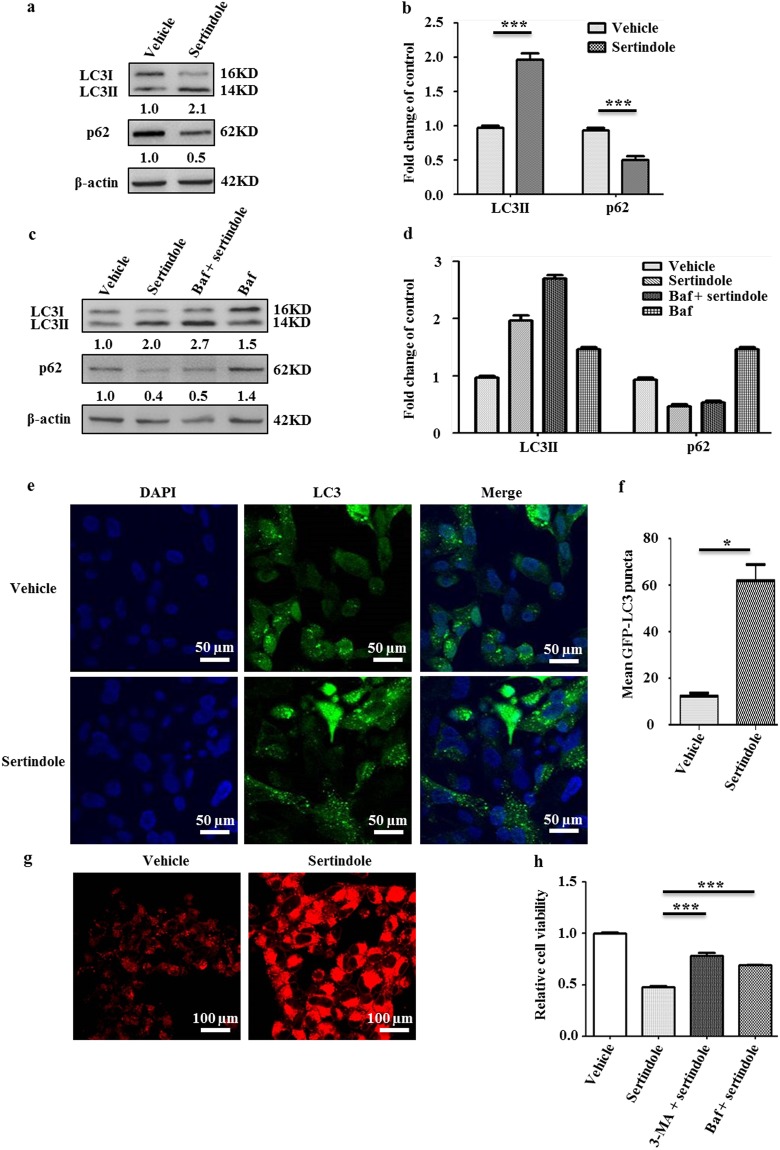
Sertindole induces cell-lethal autophagy in SUM159 cells. Detection of LC3 conversion and p62 by western blotting in cells treated with (**a**,**b**) 10 µM sertindole for 24 h, (**c**,**d**) 10 µM sertindole for 23 h and then cotreated with 10 nM baf (bafilomycinA1) for additional 1 h. β-actin was internal control for normalization. Gray intensity was analyzed by Quantity One. Error bars represented the mean of triplicates ± S.D. (**e**,**f**) GFP-LC3 puncta dots in cells incubated with vehicle or 10 µM sertindole for 2 h. Green, GFP-LC3. Blue, nuclei. Scale bars, 50 µm. The puncta dots of 50 cells in three randomly-selected fields were scored. (**g**) Lysosomes of cells treated with vehicle or 10 µM sertindole for 2 h. Red, lysosomes. Scale bars, 100 µm. (**h**) Relative viability of cells treated with 20 µM sertindole alone vs. cotreated with either 1 mM 3-MA (3-methylade) or 10 nM baf (bafilomycinA1) for 36 h. Cell viability was measured by MTT assay. Error bars represented the mean of triplicates ± S.D. *P < 0.1, ***P < 0.01.

To elucidate the basis for sertindole anti-cancer efficacy, we examined sertindole actions in SUM159 cells. Because a previous study had demonstrated that sertindole modulated lethal autophagy of neuroblastoma-derived SH-SY5Y cells^32^, we next examined autophagy and cell viability of sertindole-treated TNBC cells. Autophagy is an intracellular degradation system consisting of multiple steps characterized by increasing accumulation of autophagic structures including autophagosomes and autolysosomes^33^. Here, we first measured autophagosome levels by detecting formation of LC3II from LC3I. As a result, after 24 hours of treatment, sertindole elevated LC3II conversion significantly (*P* < 0.01) (Fig. 2a,b). It’s known that autophagic flux is often used to evaluate autophagic degradation activity^33,34^. Here we employed p62 as a marker to monitor the autophagic flux in sertindole-treated cells, due to this protein that serves as a bridge between ubiquitinated proteins and autophagic machinery involved in lysosomal degradation^33,34^. Subsequently, we observed a significant reduction in the level of p62 (*P* < 0.01), indicating the occurrence of autophagic flux in this process (Fig. 2a,b). Next, cotreatment with bafilomycinA1 (baf), a late-phase autophagy inhibitor preventing fusion between autophagosomes and lysosomes^35^, was conducted to further enable confirmation of autophagic flux. As a result, LC3II and p62 both exhibited increased accumulation in cotreated cells vs. cells treated with sertindole alone (Fig. 2c,d). Moreover, sertindole incubation also increased the mean number of GFP-LC3 puncta dots in cells by ~5.0-fold (Fig. 2e,f, Supplementary Fig. S2). Because substrates in autophagosomes are delivered to lysosomes for degradation after autophagy is induced^33,34^, lysosomal activity was also monitored to further delineate the activation of autophagic flux. Ultimately, detection of lysosomes using Lyso Tracker dye demonstrated an increased density of lysosomes in sertindole-treated cells (Fig. 2g).

The role of autophagy is complex and it can either favor cell survival or death^36–39^. To explore the action of autophagy in sertindole-treated SUM159 cells, we respectively inhibited the autophagy system using inhibitors 3-methylade (3-MA)^40^ and baf, and then measured cell viability. Blockage of autophagy by 3-MA or baf increased cell viability by averages of 62.3% and 53.8%. Therefore, sertindole-induced autophagy must rely on a cell-lethality mechanism in sertindole action (Fig. 2h).

**Figure 3:**
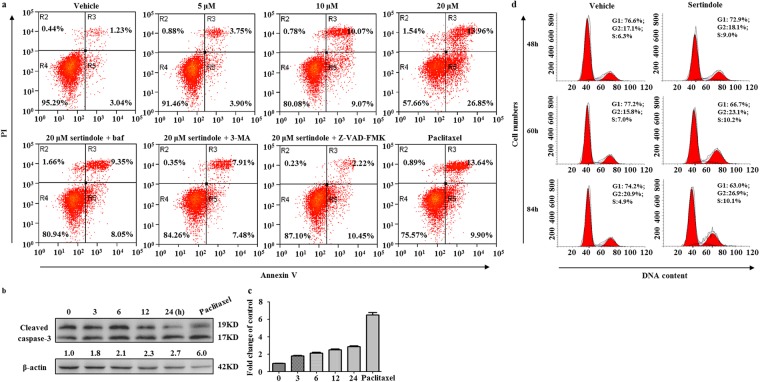
Sertindole induces apoptosis in SUM159 cells which is autophagy-associated. (**a**) Apoptosis detected by flow cytometry in cells treated with sertindole alone or cotreated with either 50 μM Z-VAD-FMK, 1 mM 3-MA (3-methylade) or 10 nM baf (bafilomycinA1) for 36 h. n = 3. (**b**,**c**) Cleaved caspase-3 was detected in cells treated with 10 μM sertindole at 0, 3, 6, 12 and 24 h post treatment by western blotting. 10 nM paclitaxel treating cells for 24 h was included as positive control. β-actin was internal control for normalization. Gray intensity was analyzed by Quantity One. Error bars represented the mean of triplicates ± S.D. (**d**) Cell cycle was detected in cells treated with vehicle or 10 μM sertindole for 48, 60 and 84 h by flow cytometry. n = 3.

Cell-lethal autophagy can depend on apoptosis^41^. We therefore measured apoptosis in sertindole-treated SUM159 cells using flow cytometry. As sertindole is a fluorescent chemical, to determine whether fluorescence of itself interfered with the measurements, we first measured the fluorescence of sertindole-incubated cells in the absence of additional fluorescent staining. Subsequently, barely any positive signal was detected, demonstrating that sertindole itself did not influence fluorescence measurements in the flow cytometry (Supplementary Fig. S3). As a result of apoptosis detection in sertindole-treated cells, a dose-dependent increase of apoptosis was observed after 36 hours of treatment (Fig. 3a). Moreover, we found that Z-VAD-FMK, an inhibitor of caspase family proteins, rescued 92.3% of cells from apoptosis and death, implicating apoptosis as the cause of cell death (Fig. 3a). Furthermore, to understand the role of autophagy in apoptosis, we blocked autophagy using inhibitors 3-MA and baf in sertindole-treated cells. Subsequently, cotreatment with 3-MA or baf reduced apoptosis in sertindole-treated cells by 65.9% and 44.9% respectively, thus implicating decreased autophagy led to decreased apoptosis and the apoptosis was autophagy-associated (Fig. 3a). Concurrently, we also measured cleaved caspase-3 levels by western blotting, which was increased after 3 hours of sertindole treatment (Fig. 3b,c). Notably, evidence of autophagy occurred as early as 2 hours after treatment with 10 µM sertindole, as demonstrated by increased GFP-LC3 puncta dots formation (Figs 2e,f and S2). However, apoptosis reflected by appreciable caspase-3 cleavage was not a significant finding until 3 hours post treatment (Fig. 3b,c). Annexin-V-based detection of apoptosis was even not significant after 24 hours of treatment (Supplementary Fig. S4). The earlier timing of autophagy than apoptosis therefore served as further evidence that autophagy might actively induce apoptosis.

In order to rule out another possible mechanism for cell death, mitotic catastrophe^42^, we investigated cell cycle in sertindole-treated cells. Our results demonstrated that only a minor percentage (15.1%) of G1 phase loss was observed after 84 hours of treatment (Fig. 3d). Therefore, the later timing of the appearance of mitotic catastrophe indicated it might be a downstream outcome of autophagy or apoptosis and therefore was probably not directly relevant to causation of cell death in this study.

### Overexpression of 5-HT6, a receptor bound directly by sertindole, suppresses sertindole effects

**Figure 4:**
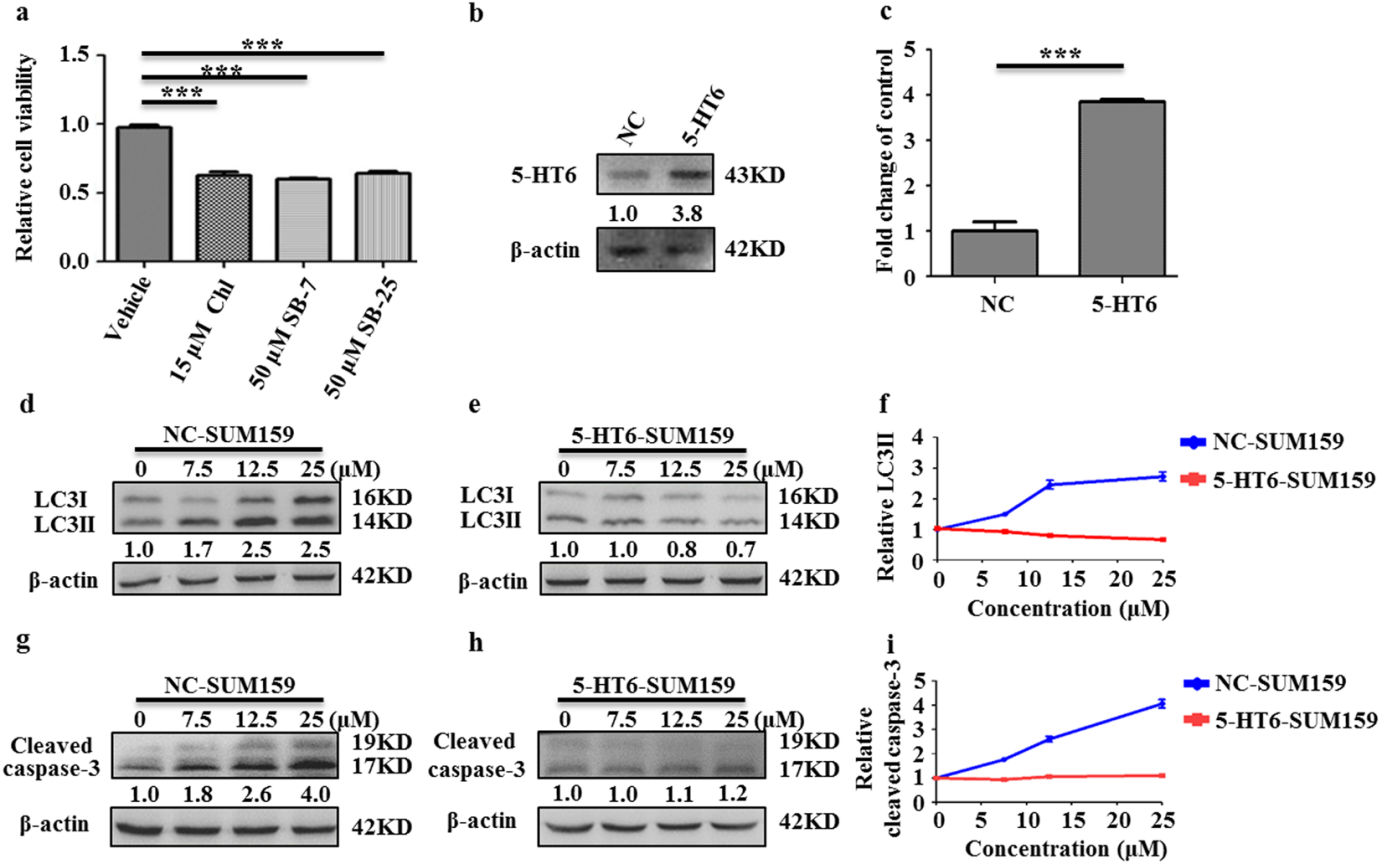
Overexpression of 5-HT6 receptor suppresses sertindole-induced LC3 conversion and caspase-3 cleavage in SUM159 cells. (**a**) Relative viability of cells treated with vehicle only vs. 5-HT6 target-specific inhibitor chlorprothixene (Chl), SB742457 (SB-7) or SB258585 (SB-25) for 48 h by MTT assay. Error bars represented the mean of triplicates ± S.D. ***P < 0.01. (**b**,**c**) Detection of 5-HT6 in surface membranes of SUM159 cells by western blotting. NC represented control SUM159 cells (NC-SUM159); 5-HT6 represented 5-HT6-overexpressing SUM159 cells (5-HT6-SUM159). Error bars represented the mean of triplicates ± S.D. ***P < 0.01. (**d**–**f**) LC3 conversion detected in NC-SUM159 cells and 5-HT6-SUM159 cells treated with various concentrations of sertindole for 24 h. (**g**–**i**) Cleaved caspase-3 detected in NC-SUM159 cells and 5-HT6-SUM159 cells treated with various concentrations of sertindole for 24 h. β-actin was internal control for normalization. Gray intensity was analyzed by Quantity One. Error bars represented the mean of triplicates ± S.D.

Previous work had demonstrated that sertindole-induced elevation of reactive oxygen species (ROS) was associated with the cell-lethal autophagy^32^. However, the directly-reacting targets of sertindole are still not clear. 5-HT2A, 5-HT2C, 5-HT6 and D2R receptors are known as sertindole’s directly-bound antagonistic targets from the previous literatures^43,44^. These receptors are G-protein-coupled receptors (GPCRs). Inhibition of such GPCRs results in subsequent inhibition of downstream receptor-coupled G proteins, which have been thought to be a reason for autophagy induction^45–47^. Moreover, reports have consistently suggested that inhibition of GPCRs bears a close relationship with effects on cell proliferation, survival and tumorigenesis^48–50^. Therefore, we considered that these sertindole-targeted GPCRs might have an involvement in the autophagic cell death caused by sertindole. Subsequently, we screened various sertindole-binding receptors. As a screening method, we first inhibited activity of these GPCRs using target-selective antagonists and monitored whether GPCRs inhibition resulted in a lower cell viability, as observed for sertindole. After conducting experiments using antagonists of 5-HT2A (ketanserin), 5-HT2C (agomelatine), 5-HT6 (SB271046) and D2R (levosulpiride), only SB271046, the antagonist of 5-HT6, induced cytotoxicity (Supplementary Fig. S5). To confirm this observation, we further tested three additional 5-HT6 antagonists, SB258585 (SB-25), SB742457 (SB-7) and chlorprothixene (Chl), and demonstrated that all of these antagonists induced lower cell viability as well (Fig. 4a). Consistent with previous studies showing that activation of 5-HT6 promoted proliferation of neurites and restrained apoptosis^51,52^, the work described here revealed that inactivation of 5-HT6 attenuated proliferation of SUM159 cells. Moreover, 5-HT6 is one G_s_-protein-coupled receptor elevating cAMP production^51,52^. Inhibition of G_s_-cAMP signaling can induce autophagy^53,54^. All these lines of evidence promoted us to further explore the role of 5-HT6 receptor in sertindole effects.

To determine whether sertindole decreases cell proliferation via 5-HT6 antagonism as the mechanism of action, we first demonstrated that 5-HT6 protein was indeed present in breast cancer SUM159 cells. As a result, we observed an expression of 5-HT6 in the membranes of SUM159 cells (Fig. 4b,c). To understand the role of 5-HT6 receptor in sertindole-induced autophagy and apoptosis, we further established 5-HT6-overexpressing SUM159 cells (represented by 5-HT6-SUM159 and the control cells represented by NC-SUM159), and investigated LC3II and cleaved caspase-3 accumulation in both cells after sertindole treatment. With increasing sertindole concentration, both LC3II accumulation and a higher level of caspase-3 cleavage was observed in NC-SUM159 cells (Fig. 4d,g). By comparison, in sertindole-treated 5-HT6-SUM159 cells, LC3II formation was not increased and even exhibited a slight reduction (Fig. 4e,f). This result indicated that 5-HT6 overexpression entirely blocked sertindole-triggered autophagosome formation. Furthermore, caspase-3 cleavage was also insensitive to sertindole treatment, suggesting that apoptosis was suppressed by overexpression of 5-HT6 as well (Fig. 4h,i).

### Inactivation of 5-HT6 by antagonist SB258585 augments sertindole effects

**Figure 5:**
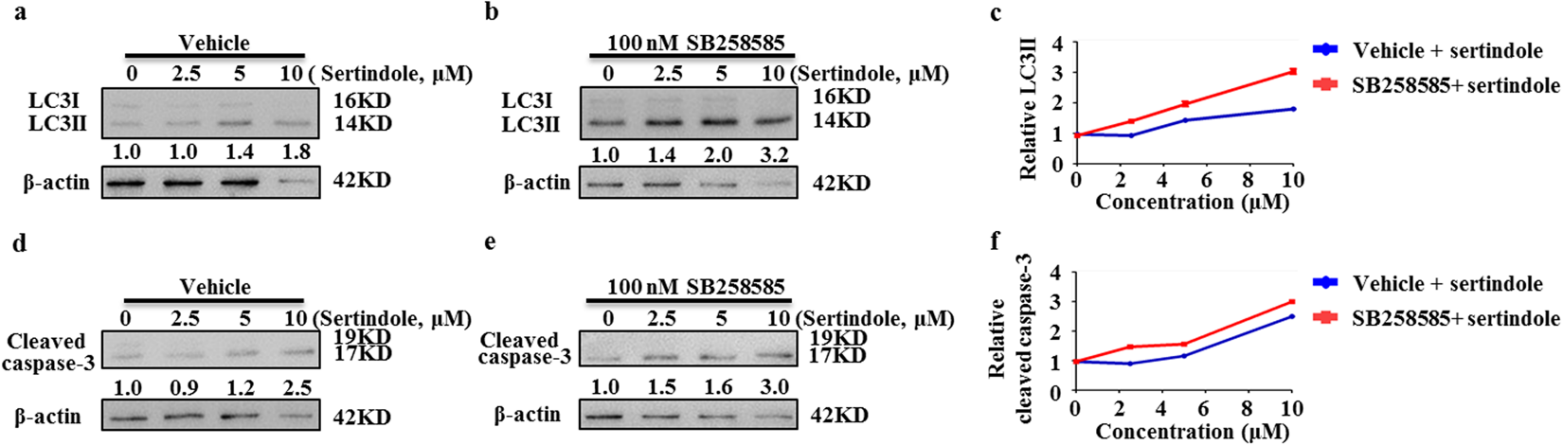
Inactivation of 5-HT6 receptor by its target-specific antagonist SB258585 augments LC3II conversion and caspase-3 cleavage in sertindole-treated SUM159 cells. (**a**–**c**) LC3 conversion and (**d**–**f**) Cleaved caspase-3 was detected in cells treated with sertindole alone, or cotreated with additional 100 nM SB258585 for 24 h. β-actin was internal control for normalization. Gray intensity was analyzed by Quantity One. Error bars represented the mean of triplicates ± S.D.

On the other hand, to ensure that 5-HT6 receptor inhibition is involved in sertindole-induced cell autophagy and apoptosis, we also used a 5-HT6 specific antagonist SB258585 to inactivate the 5-HT6 receptor and then monitored downstream sertindole-induced effects. In cells treated with sertindole alone, LC3 and cleaved caspase-3 exhibited no alteration at the concentration of 2.5 µM (Fig. 5a,d). However, when cells were cotreated with sertindole and additional 100 nM SB258585, 2.5 µM sertindole was sufficient to elevate the levels of LC3II and cleaved caspase-3 in cells (Fig. 5b,e). 100 nM SB258585 also augmented sertindole-induced LC3II conversion and caspase-3 cleavage at sertindole concentrations of 5 µM and 10 µM (Fig. 5b,c,e,f). These results, coupled with the observation that overexpression of 5-HT6 could block sertindole-induced LC3II and cleaved caspase-3 accumulation, indicated that sertindole-caused autophagy and apoptosis might rely on a 5-HT6 inhibition mechanism.

### An increased expression in pre-invasive breast tumor tissue and 5-HT6 may have an association with cell proliferation

**Figure 6:**
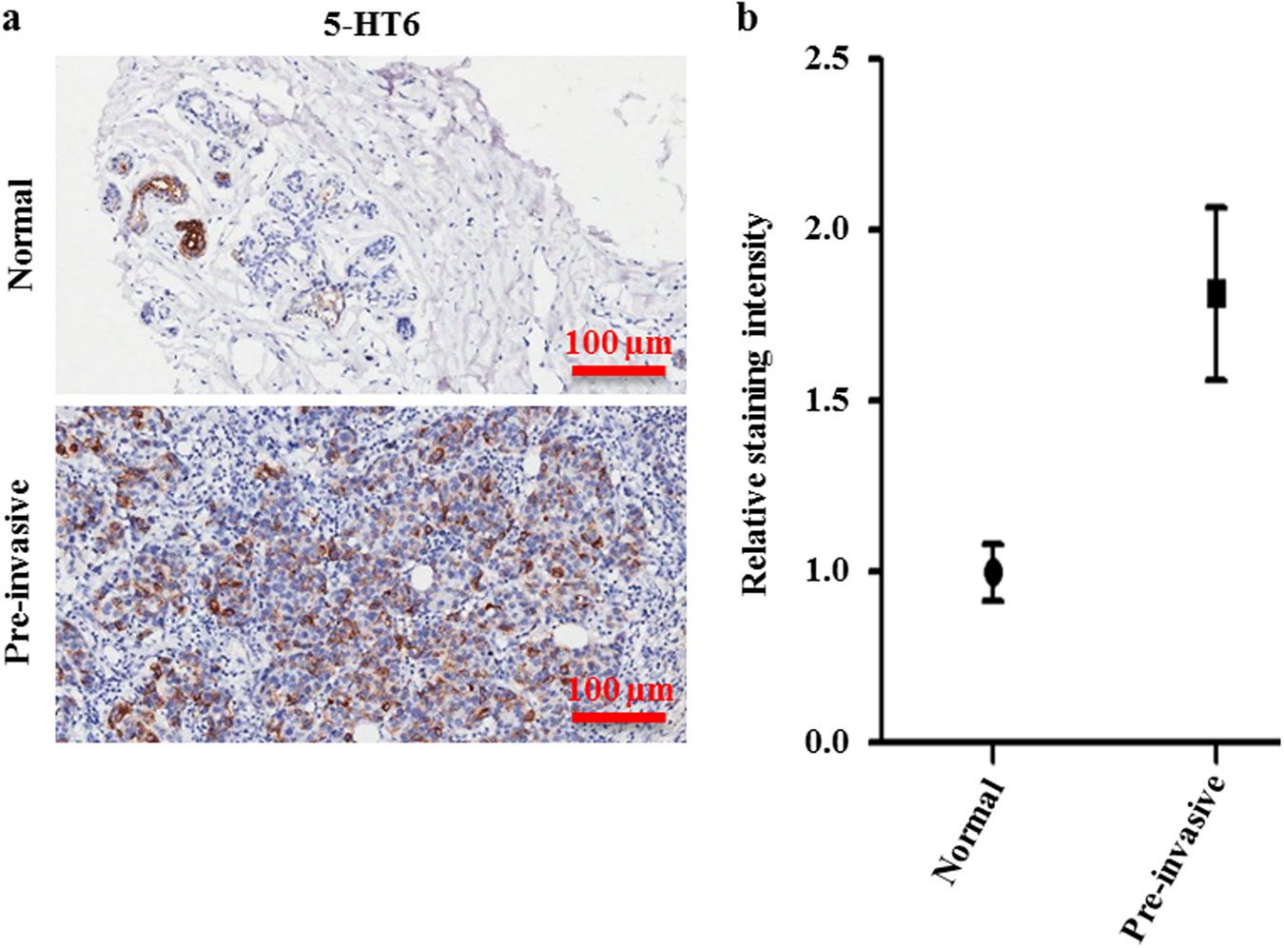
5-HT6 receptor expresses twofold greater in pre-invasive breast tumor tissue than normal breast tissue. (**a**) 5-HT6 receptor was detected in human normal breast tissue and pre-invasive breast tumor tissue by tissue array. Scale bars, 100 µm. (**b**) Statistic-graph of relative staining intensity of 5-HT6 receptor in human normal breast tissue vs. pre-invasive breast tumor tissue.

The 5-HT6 receptor had been previously reported to promote growth of neurites^51^. Because our results demonstrated that 5-HT6 inhibition led to attenuated SUM159 cell proliferation, we considered that 5-HT6 receptor possibly had a function in proliferation of breast tumor. To explore its role in breast cancer, we observed 5-HT6 expression in human breast tumor tissues and normal breast tissue, and observed twofold greater 5-HT6 expression in pre-invasive breast tumor tissue vs. normal breast tissue (Fig. 6a,b). This result implied that 5-HT6 receptor was increasingly expressed in human pre-invasive breast cancer, and further, probably had an association with cell proliferation.

### Attenuation of MDA-MB-231 orthotopic tumors by sertindole

**Figure 7:**
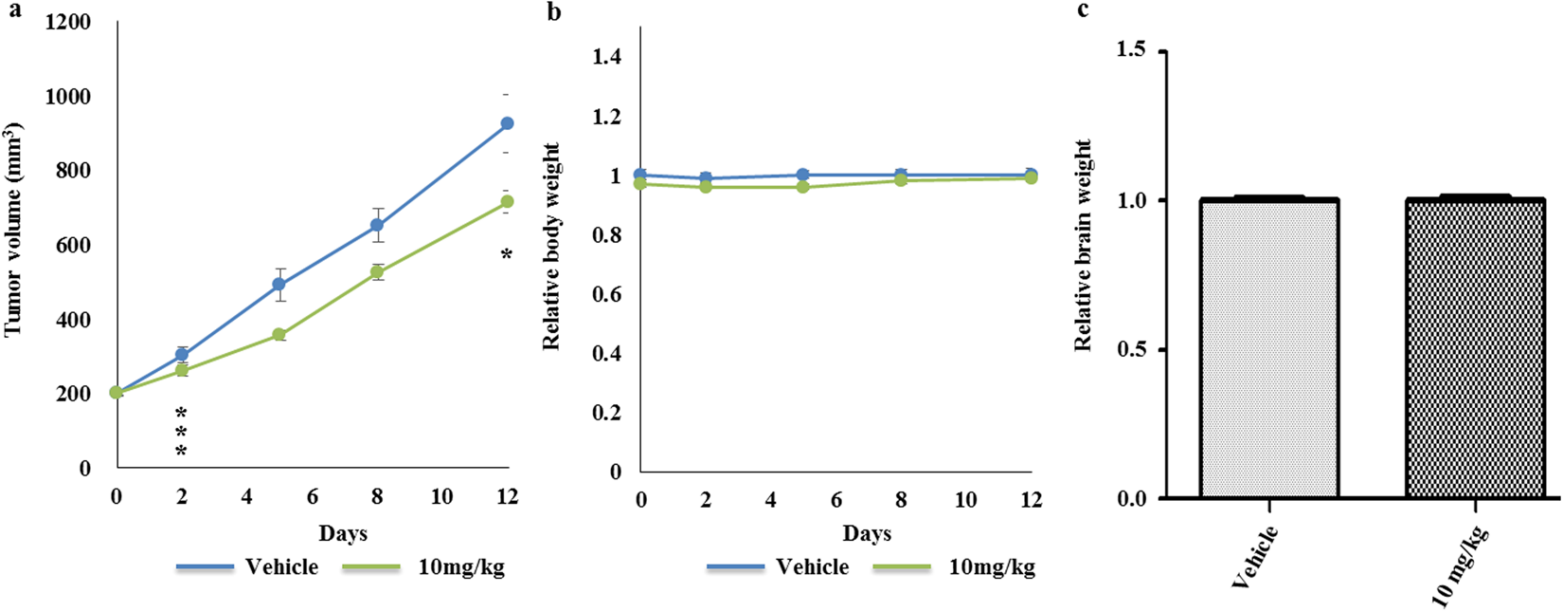
Sertindole suppresses growth of MDA-MB-231 tumors in xenografted mice. (**a**) Tumor-growth curve of xenografted mice. (**b**) Body-weight curve of xenografted mice. (**c**) Brain weights of xenografted mice. Mice were received vehicle or 10 mg/kg sertindole for 12 days. Error bars represented the mean of six ± S.D. *P < 0.1, ***P < 0.01.

Based on the *in vitro* results described in this work, sertindole is a promising agent for treatment of TNBC. To test the efficacy of sertindole *in vivo*, we implanted MDA-MB-231 human TNBC cells exhibiting strong metastatic tendencies, orthotopically into the right mammary fat pads of immune-deficient Balb/c mice. Next, the mice received a daily treatment of 10 mg/kg sertindole by oral gavage, a dosage of about twofold the maximal therapeutic antipsychotic dose for humans^55^. As a result, the average tumor volume of sertindole-fed mice exhibited a 22.7% reduction in size after a 12-day administration regimen (Fig. 7a). This result demonstrated that sertindole possessed antitumor activity *in vivo*.

### Sertindole treatment elicits no apparent reduction of the body and brain weights in mice with tumor burden

Sertindole is a clinically applied drug with established safe therapeutic dosage ranges for the treatment of psychosis. However, the applicability of antipsychotic therapeutic doses for cancer models is still unclear. Therefore, we monitored relevant characteristics, including body and brain weights, of immune-deficient Balb/c mice after MDA-MB-231 tumor implantation with or without daily sertindole gavage treatment. After a 12-day treatment period, the body weights of mice fed 10 mg/kg sertindole were indistinguishable from control mice (Fig. 7b). Furthermore, the brain weights of both groups of mice exhibited no significant difference (Fig. 7c).

### Antiproliferative activities of sertindole against various cancer cell lines

**Figure 8:**
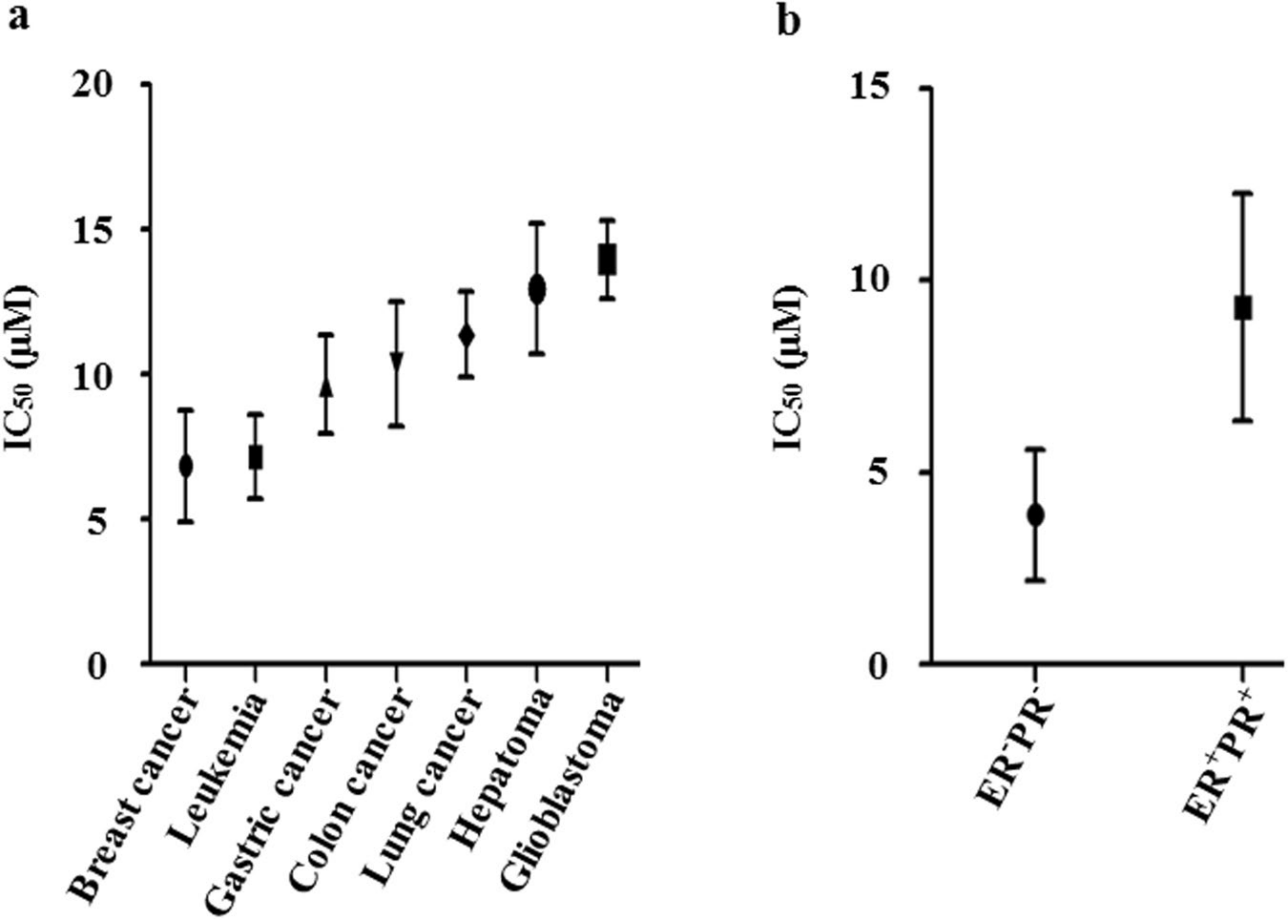
Sertindole impairs proliferation of many cancer cell lines *in vitro*, especially ER^−^PR^−^ breast cancer cells. (**a**) Statistics-graph of IC_50_ values of sertindole toward seven cancer types including 29 cell lines. (**b**) Statistics-graph of IC_50_ values of sertindole toward ER^−^PR^−^ breast cancer cell lines (MDA-MB-453, MDA-MB-231, SUM159) vs. ER^+^PR^+^ breast cancer cell lines (MCF-7, T47D, ZR-75-1). All these cells were treated with sertindole for 48 h. The IC_50_ values were measured by MTT assay.

To determine the antitumor spectrum of sertindole effectiveness, we assessed the toxicity of sertindole toward 29 cell lines spanning a total of seven cancer types (Supplementary Table S2). Among these cell lines, breast cancer and leukemia lines showed the highest sensitivity to sertindole, with IC_50_ concentrations ranging between 0.8–12.7 µM and 2.7–4.6 µM, respectively, while hepatoma and glioblastoma lines exhibited moderate sensitivity, ranging between 12.7–15.3 µM and 8.6–16.1 µM, respectively (Fig. 8a). These results demonstrated that sertindole was effective toward many cancers *in vitro*. Furthermore, among breast cancer cell lines, we found that ER^−^PR^−^ cell lines were more sensitive to sertindole than ER^+^PR^+^ cell lines. Specifically, the average IC_50_ value of ER^−^PR^−^ cell lines was 4.1 µM (MDA-MB-453, MDA-MB-231, SUM159) compared with the average IC_50_ value of 9.3 µM observed for ER^+^PR^+^ cell lines (MCF-7, T47D, ZR-75-1) (Fig. 8b).

## Discussion

**Figure 9:**
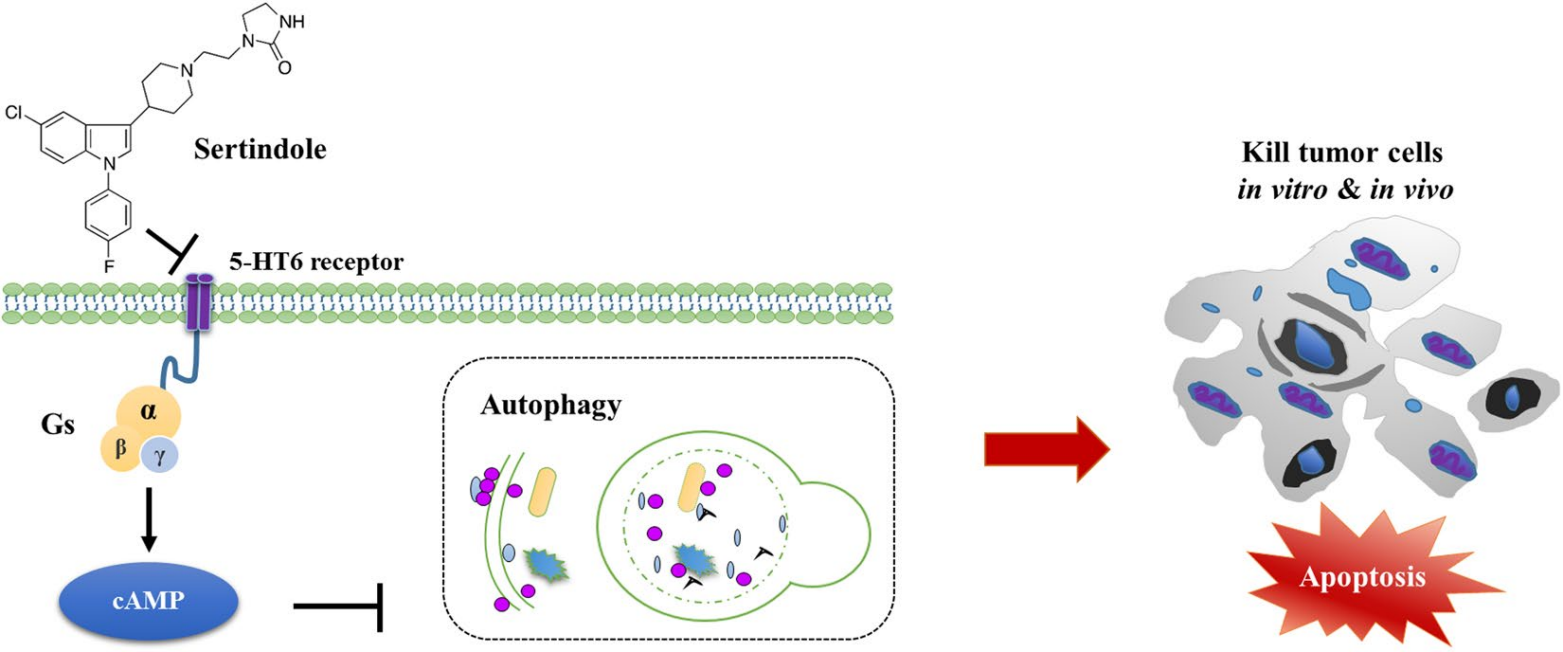
Sertindole kills tumor cells *in vitro* and *in vivo* through autophagy-associated apoptosis and 5-HT6 receptor partly mediates this process. During this process, sertindole directly binds and inactivates cell surface receptor 5-HT6. 5-HT6 receptor is one G_s_-protein-coupled receptor, inhibition of which leads to reduced production of cAMP. Down regulation of cAMP has been demonstrated to cause autophagy. With accumulated autophagic stress in sertindole-treated cells, apoptosis happens following the autophagy when the stress crosses threshold. At the end, apoptosis leads cells to die.

Our current study demonstrates for the first time both *in vitro* and *in vivo* antitumor effects of the SGA drug sertindole towards TNBC, a type of currently untreatable breast cancer that commonly metastasizes to the brain. Our results revealed that sertindole treatment caused cytotoxicity via autophagy-associated apoptosis, in which the interaction of sertindole with the 5-HT6 receptor might play an important role. Because sertindole also attenuated the growth of metastatic breast tumors *in vivo*, this study revealed the impressive potential of sertindole as a chemotherapeutic agent against metastatic TNBC, with a probably particular application of breast-to-brain metastases (Fig. 9).

For a range of typical therapeutic dosages, mortality of sertindole-treated schizophrenic patients from all causes is far lower than FGA drugs^24–28,56–58^, and equal to that of patients receiving SGA drugs risperidone and olanzapine^59,60^. Moreover, sertindole induces less severe and fewer extrapyramidal symptom (EPS)-related adverse events than do FGAs^26,30,56–58,61^. Notably, EPS events triggering several conditions, such as tardive dyskinesia, can result in increased mortality^26^. Although the reasons underlying the fewer spontaneous deaths of SGAs vs. FGAs patients are not yet known, EPS-related events may play a role and further justify choices regarding drugs applied clinically. In rats, the administration of up to 40 mg/kg sertindole, dose that is 10-fold greater than the maximal therapeutic dose of psychosis, does not elicit EPS^62^. In human, the EPS-triggering dose threshold may even approach 800 mg, 33 times the maximal antipsychotic therapeutic dose^44,62^. Furthermore, compared to FGAs inducing strongly increased deaths in elderly people, age as a factor in the mortality rate of sertindole-treated patients does not appear to be significantly involved. Demonstrated by an independent large-sample study, in all sertindole-related deaths, people older than 45 years of age only accounts for 16.7% of the total numbers, suggesting that sertindole may not cause more deaths in elderly people^57^. All these lines of evidence underscore the potential of repurposing sertindole as an antitumor agent for clinical treatment.

Cho and colleagues have reported that sertindole elevated reactive oxygen species (ROS) to induce cell-lethal autophagy in SH-SY5Y cells^32^. Our study here further-in-depth indicates that autophagy is first induced as a primary response that subsequently induces apoptosis to trigger cell death in sertindole-treated SUM159 cells. In such autophagy-preceding-apoptosis cases, to adapt high level of stress, autophagy is immediately induced to digest damaged elements in cells; next, apoptosis will be stepwise irreversibly activated for dismantling aberrant cells apart from the population^63–66^. Furthermore, our study reveals that 5-HT6, a directly-bound receptor of sertindole, may be involved in sertindole-induced autophagic cell death. Specifically, overexpression of 5-HT6 suppressed autophagy and apoptosis induced by sertindole, as demonstrated by the reduction of LC3II and cleaved caspase-3, and inactivation of 5-HT6 receptor augmented sertindole-induced effects. Interestingly, one effector in 5-HT6/cAMP signaling, Epac, is a suppressor of ROS production^67–70^, suggesting inhibition of 5-HT6 in sertindole-treated cells may positively modify ROS levels, thus reconciling Cho’s results and our observations.

Regarding the mechanism of sertindole action, the involvement of 5-HT6 receptor can be further supported using several lines of evidence. First, 5-HT6 has been observed to act through stimulation of cell proliferation, as demonstrated in studies showing that 5-HT6 stimulates cell growth of neurites through activation of Cdc42^51^. 5-HT6 also activates some other proliferation-promoting proteins, such as mTOR and Jab-1^52,71^. Additionally, in monoamine oxidase A (MAO-A) knockout mice, 5-HT6 favors cell survival and thus restrains apoptosis of embryonic brain cells^72^. Cells injured by toxins have been shown to exhibit increased expression of 5-HT6, implying a protective role of this receptor^52^. Moreover, previous studies have revealed that 5-HT, a natural ligand of 5-HT6, is present in many tissues and has been demonstrated to correlate closely with cell proliferation, survival and tumor progression^73–77^. In agreement with these aforementioned reports, the results of our study demonstrates that sertindole inhibition of 5-HT6 action causes reduced cell viability and induction of apoptosis in SUM159 cells. Also consistent with previous studies, the results of this work further demonstrate in humans a higher detection of 5-HT6 in pre-invasive breast cancer tissue than in normal breast tissue.

As a final note, sertindole may exhibit more promising efficacy toward breast-to-brain metastases (BTBM). First, due to its ability to penetrate the BBB with a brain/plasma ratio larger than 1.2, sertindole reaches a higher concentration in brain than in plasma^78^. Moreover, as reported for drugs such as penfluridol and vorinostat, anti-BrM efficacy frequently tends to be 20–60% higher than efficacy against primary tumors outside of the brain^79,80^. Further studies will focus on the anti-BTBM effect of sertindole to evaluate its promise as a repurposed drug for BTBM treatment.

## Methods

### Cell culture

U251, A172, U87-MG, U118-MG, HT-29, SW480 and HepG2 cells were cultured in DMEM (Cellgro); CCRF-CEM, K562, Jurkat, NCI-H460, A549, NCI-H446, NCI-H661, 801-D, AGS, MKN45, BGC-823, SGC-7901, COLO205, HCT-15, Bel-7402, T47D and ZR-75-1 cells were grown in RPMI-1640 (Cellgro); SW620, MDA-MB-231 and MDA-MB-453 cells were cultured in L-15 (Cellgro); MCF-7 cells were maintained in DMEM supplied with 0.01 mg/ml insulin; SUM159 cells were grown in DMEM/F12 (Cellgro) supplied with 0.05 mg/ml insulin and 0.01 mg/ml hydrocortisone; MCF-10A cells were cultured in DMEM/F12 supplied with 100 ng/ml cholera toxin. All cell lines were maintained in media with 10% fetal bovine serum (FBS) (Cellgro) at 37 °C in a humidified atmosphere. SW620, MDA-MB-231 and MDA-MB-453 cells were cultured without CO_2_ and the rest of cells were cultured with 5% CO_2_. MCF-10A cell line was purchased from American Type Culture Collection. SUM159 cell line was kindly donated by Pro. Guo group (Tsinghua University, China). The rest of cell lines were purchased from Jiete Biology Company.

### Chemicals

Asenapine (S1283), blonanserin (S2112), droperidol (S4096), ziprasidone (S1444), iloperidone (S1483), amitriptyline (S3183), SB271046 (S2856), ketanserin (S2232), agomelatine (S1243), levosulpiride (S2104), SB742457 (S2894) and chlorprothixene (S1771) were purchased from Selleck. 3-methylade (5142-23-4) was purchased from Energy Chemical. Clozapine (C6305), SB258585 (S1194) and bafiloycinA1 (B1793) were purchased from Sigma. Sertindole (106516-24-9) was purchased from UHN.

### Cytotoxicity studies

Cells were seeded into 96-well plates at a density of 3,000 to 5,000 cells/well and incubated with 5% CO2 at 37 °C for 24 h before drug treatment. Drugs diluted by culture medium at indicated final concentrations were added into the culture medium and cells were incubated for 36 h or 48 h. After incubation, cell viability was determined by MTT assay. In brief, MTT solution was added to each well at a terminal concentration of 0.5 mg/ml. Subsequently, the plates were incubated at 37 °C with 5% CO_2_ for 4 h. Discarded the culture medium and replaced it with 100 µl 100% DMSO. Plates were incubated at room temperature in dark for 2 h. Absorbance was read at 490 nm and the IC_50_ values were then calculated.

### Colony formation assay

Cells were seeded into a 6-well plate at a density of 300 to 500 cells/well and cultured for 24 h before treatment. Then the culture medium was substituted with fresh medium containing sertindole with replacement of culture medium every three days and incubated for 15 days. The numbers of colonies were counted after fixed with methanol for 15 min and stained with Giemsa for 30 min.

### Wound healing assay

8 × 10^5^ to 10^6^ cells/well were seeded into a 6-well plate. After 24 h culture, three scratches into the confluent cell layers were created by a sterile p50 micropipette tip. The cell layers were washed with PBS for three times to remove the floating cells and debris. Cells were then cultured in fresh basic culture medium without serum for 72 h. The images of the wounds were acquired at 0 h and 72 h after scratching with a Leica CTR4000 system mounted on a phase-contrast microscope using software of LAS version 4.6. The distances moved by the cells were calculated by measuring the wound widths at 72 h and then were subtracted by the wound widths at 0 h (Width_migration_ = Width_0 h_ − Width_72 h_). The values were expressed by migration percentages and the wound widths at 0 h were set as 0%.

### Invasion assay

10^5^ to 10^6^ cells were suspended with 0.1 ml serum-free culture medium and seeded in an upper chamber of a 24-well insert (Corning, 3422). The insert was put in a 24-well plate with 1 ml 10% FBS culture medium in the lower chamber and the cells were cultured for 24 h. Subsequently, the noninvasive cells on the upper chamber were removed with cotton swabs. The invasive cells on the lower sides of the membranes were then fixed with 4% paraformaldehyde for 15 min, washed with PBS twice and stained with trypan blue for 12 h. The cells were washed with PBS for three times and dried in air. The lower sides of the membranes were photographed randomly in three fields of views with a Leica CTR4000 system mounted on a phase-contrast microscope using software of LAS version 4.6. Numbers of cells in each field were recorded.

### Immunofluorescence

3,000 to 5,000 cells were seeded on a poly-_L_-lysine-coated glass coverslip laying in a 35 mm dish before 24 h of treatment. After treatment, cells were fixed with 4% paraformaldehyde for 15 min at room temperature. Subsequently, cells were incubated in permeabilized and blocking buffer (3% BSA in PBS + 0.5% Triton X-100) at room temperature for 1 h. After the blocking, the cells were probed with FITC-phalloidine (Beyotime, C1033; 1:50) at room temperature for 1 h. The cells were washed softly with PBS for three times and mounted with DAPI staining buffer (Beyotime, C1005) for nuclei visualization. Images of the cells were acquired by a Leica CTR4000 system mounted on a phase-contrast microscope using software of LAS version 4.6.

### Preparation of membrane protein

Membrane fractions were prepared by using Membrane and Cytoplasmic Protein Extraction Kit (Beyotime, P0033) according to the manufacturer’s instructions.

### Flow cytometry

In apoptosis analysis, cells were stained using Annexin V/Dead Cell Apoptosis Kit (Beyotime, C1062) according to the manufacturer’s instructions. Briefly, harvested cells were suspended with Annexin V/PI staining buffer (195 µl Annexin-V binding buffer + 5 µl Annexin V-FITC + 10 µl PI) and incubated in dark for 20 min at room temperature. Stained cells were filtered into single-cell suspension and then analyzed by MoFlo^TM^ XDP flow cytometer using software of Summit V5.2. In cell cycle analysis, harvested cells were fixed with 75% ethanol at −20 °C for 12 h. The fixed cells were softly washed by ice-cold PBS twice and then suspended with PI staining buffer (20 µg/mL PI, 200 µg /mL RNase, 0.1% Triton-X 100), placing in dark at room temperature for 30 min. Cells were filtered into single-cell suspension and then analyzed by MoFlo^TM^ XDP flow cytometer using software of Summit V5.2.

### Orthotopic breast tumor model and sertindole treatment

Female Balb/c immune-deficient mice (6–8 weeks old) were purchased from Crown Bioscience Inc. in China. All mice were implanted with 10^7^ MDA-MB-231 cells mixed with an equal volume of Matrigel^TM^ basement membrane matrix (Corning) into the right fat pad. Tumor volumes were calculated according to the following formula *V* = {(*Length*) × (*Width*)}^2^*/2*. When tumor volumes reached ~200 mm^3^, mice were distributed randomly into experimental groups and then treated with intragastric administration of vehicle or sertindole once per day. Tumor volumes were measured (mean values and 95% confidence intervals) two times each week. All mouse studies were approved by the Association for Assessment and Accreditation of Laboratory Animal Care (AAALAC) and conducted by adherence to AAALAC guidelines involving animal experiments.

### Statistical analysis

Prism 6.0 software was used for statistical analysis (GraphPad Software, Inc.). All results were shown as means ± standard deviation or as the error of means. Statistical significance was assessed using the Student’s t-test. *P < 0.1, **P < 0.05, ***P < 0.01.

## Electronic supplementary material

Supplementary information

## Data Availability

All data generated or analyzed during this study are included in this published article (and its Supplementary Information files).
