# Supplementary material for: Antiproliferative activities of the second-generation antipsychotic drug sertindole against breast cancers with a potential application for treatment of breast-to-brain metastases
Source: Sci Rep. 2018 Oct 25;8:15753. doi: 10.1038/s41598-018-33740-0 (PMC6202417; doi:10.1038/s41598-018-33740-0)
Supplement: Supplementary file 1 — Supplementary information [file 41598_2018_33740_MOESM1_ESM.doc]

**Title page**

**Title:** Antiproliferative activities of the second-generation antipsychotic drug sertindole against breast cancers with a potential application for treatment of breast-to-brain metastases

**Authors and affiliations:** Wei Zhang^++, 1, 4^, Cun Long zhang ^++, 3^, Feng Liu^1^, Yu Mao^1^, Wei Xu^4^, Tingting Fan^1^, Qinsheng Sun^1, 4^, Shengnan He^1^, Yuzong Chen^3^, Wei Guo^*, 4^, Ying Tan^*, 1^ and Yuyang Jiang^*, 1, 2^

^1^ State Key Laboratory of Chemical Oncogenomics, the Graduate School at Shenzhen, Tsinghua University, Shenzhen 518055, P. R. China

^2^ Department of Pharmacology and Pharmaceutical Sciences, School of Medicine, Tsinghua University, Beijing 100084, P. R. China

^3^ Shenzhen Technology and Engineering Laboratory for Personalized Cancer Diagnostics and Therapeutics, Shenzhen Kivita Innovative Drug Discovery Institute, Shenzhen 518055, P. R. China

^4^ School of Medicine, Tsinghua University, Beijing 100084, P. R. China

***Corresponding authors:** Wei Guo, School of Medicine, Tsinghua University, Beijing 100084, P. R. China. Tel/Fax: +86 010 6278 2975, E-mail: [weiguo@mail.tsinghua.edu.cn](mailto:weiguo@mail.tsinghua.edu.cn); or Ying Tan, State Key Laboratory of Chemical Oncogenomics, the Graduate School at Shenzhen, Tsinghua University, Shenzhen 518055, P. R. China. Tel/Fax: +86 755 2603 2094, E-mail: [tan.ying@sz.tsinghua.edu.cn](mailto:tan.ying@sz.tsinghua.edu.cn); or Yuyang Jiang, State Key Laboratory of Chemical Oncogenomics, the Graduate School at Shenzhen, Tsinghua University, Shenzhen 518055, P. R. China or Department of Pharmacology and Pharmaceutical Sciences, School of Medicine, Tsinghua University, Beijing 100084, P. R. China. Tel/Fax: +86 755 2603 2094, E-mail: [jiangyy@sz.tsinghua.edu.cn](mailto:jiangyy@sz.tsinghua.edu.cn)

**Contributing authors:** Wei Zhang, School of Medicine, Tsinghua University, Beijing 100084, P. R. China or State Key Laboratory of Chemical Oncogenomics, the Graduate School at Shenzhen, Tsinghua University, Shenzhen 518055, P. R. China. Tel: +86 755 2603 6430, E-mail: [w-z12@mails.tsinghua.edu.cn](mailto:w-z12@mails.tsinghua.edu.cn); Cun Long zhang, Shenzhen Technology and Engineering Laboratory for Personalized Cancer Diagnostics and Therapeutics, Shenzhen Kivita Innovative Drug Discovery Institute, Shenzhen 518055, P. R. China. Tel/Fax: +86 755 2603 6430, E-mail: [zhcunl@126.com](mailto:zhcunl@126.com); Feng Liu, State Key Laboratory of Chemical Oncogenomics, the Graduate School at Shenzhen, Tsinghua University, Shenzhen 518055, P. R. China. Tel: +86 755 2603 6430, E-mail: [liu.feng@sz.tsinghua.edu.cn](mailto:liu.feng@sz.tsinghua.edu.cn); Yu Mao, State Key Laboratory of Chemical Oncogenomics, the Graduate School at Shenzhen, Tsinghua University, Shenzhen 518055, P. R. China. Tel: +86 755 2603 6430, E-mail: [maoyuyu212@163.com](mailto:maoyuyu212@163.com); Wei Xu, School of Medicine, Tsinghua University, Beijing 100084, P. R. China. Tel: +86 755 2603 6430, E-mail: [072994@163.com](mailto:072994@163.com); Tingting Fan, State Key Laboratory of Chemical Oncogenomics, the Graduate School at Shenzhen, Tsinghua University, Shenzhen 518055, P. R. China. Tel: +86 755 2603 6430, E-mail: [ftt15@mails.tsinghua.edu.cn](mailto:ftt15@mails.tsinghua.edu.cn); Qinsheng Sun, School of Medicine, Tsinghua University, Beijing 100084, P. R. China or State Key Laboratory of Chemical Oncogenomics, the Graduate School at Shenzhen, Tsinghua University, Shenzhen 518055, P. R. China. Tel: +86 755 2603 6430, E-mail: sunqinsheng@163.com； Shengnan He, State Key Laboratory of Chemical Oncogenomics, the Graduate School at Shenzhen, Tsinghua University, Shenzhen 518055, P. R. China. Tel: +86 755 2603 6430, E-mail: [eheshengnan@163.com](mailto:eheshengnan@163.com); Yuzong Chen，Shenzhen Technology and Engineering Laboratory for Personalized Cancer Diagnostics and Therapeutics, Shenzhen Kivita Innovative Drug Discovery Institute, Shenzhen 518055, P. R. China. Tel/Fax: +86 755 2603 6430, E-mail: phacyz@nus.edu.sg

**^++^Co-Authors:** These authors contributed equally to this work

**Supplementary data**

**Supplementary materials and methods**

**Preparation of total protein**

Cells were washed by ice-cold PBS twice. 100 µl ice-cold lysis buffer (10 mM HEPES (pH 7.9), 10 mM KCl, 1 mM EDTA, 0.1% NP-40, protease inhibitors cocktails) was added into dishes per 10^6^ cells. Lysates were incubated on ice for 30 min and then centrifuged at 20000 g, 4 °C for 10 min. The supernatant was collected and frozen at -80 °C.

**Western blotting**

10 µg denatured protein was subjected to SDS-PAGE and transferred to a PVDF membrane. Protein-loaded membranes were then blocked by blocking buffer (5% skim milk in TBST) at room temperature for 1 h and probed with primary antibodies against β-actin (Santa Cruz, sc-81178; 1:1000), LC3B (Sigma, L7543; 1:1000), 5-HT6 (Santa Cruz, sc-28962; 1:1000) or cleaved caspase-3 (CST, 9664; 1:1000) at 4 °C for 10-12 h. After incubated with the primary antibodies, the PVDF membranes were washed by TBST buffer for three times and 5 min each time. Subsequently, the membranes were probed with the second antibodies (Beyotime, A0208 or A0216; 1:3000) at room temperature for 2 h. After the incubation, the PVDF membranes were washed by TBST buffer for three times and 5 min each time. Immuno-reactive proteins were visualized using the BeyoECL Moon Super-Signal system (Beyotime, P0018FFT) and then quantified by the software of Quantity One.

**Tissue array and** **Immunohistochemistry staining**

Paraffin blocks from 70 patients were shared by the National Human Genetic Resources Sharing Service Platform of China. Age range of these patients was from 30 to 89 years old at the time of surgery (median age was 58 years old). Informed consent was received from all participants. An approval was obtained from the Taizhou Hospital Ethics Committee. The work has been carried out in accordance with The Code of Ethics of the World Medical Association involving human experiments. The immunohistochemistry staining was performed following standard methods. Briefly, paraformaldehyde-fixed and paraffin-embedded tissue sections were conducted for 5-HT6 receptor (Creative Diagnostic, DCABH-15695; 1:200) staining. Cell nuclei were visualized by DAPI. Staining scores of 5-HT6 receptor was acquired and calculated by the following procedures: intensity scores of positive staining (negative = 0, weak = 1, moderate = 2, strong = 3) multiplied proportion of positively-stained cells of interest (25% = 1, 26-50% = 2, 51-75% = 3, 76-100% = 4).

**Supplementary Figures**

**Supplementary Figure S1** Relative viability of a confluent layer of SUM159 cells toward sertindole. Cells were treated with sertindole for 48 h and the IC_50_ value was calculated by MTT assay. Error bars represented the mean of triplicates ± S.D.

**Supplementary Figure S2** Sertindole treatment increases GFP-LC3 puncta dots in SUM159 cells. Cells were treated with (a-c) vehicle or (d-f) 10 µM sertindole for 2 h. 50 cells in three randomly-selected fields were scored. Green, GFP-LC3. Blue, nuclei.

**Supplementary Figure S3** Fluorescence of sertindole itself does not interfere outcomes of fluorescent experiments of SUM159 cells. Cells were treated with sertindole for 36 h and then stained (a) without or (b) with additional fluorescent apoptosis-detection reagents. Fluorescence of cells was detected by flow cytometry. n = 3.

**Supplementary Figure S4** 10 μM sertindole treating SUM159 cells for 24 h does not trigger significant apoptosis. Cell apoptosis was detected by flow cytometry. n = 3.

**Supplementary Figure S5** SB271046, a 5-HT6 target-specific antagonist, induces cytotoxicity of SUM159 cells. 30 µM ketanserin, agomelatine, SB271046 and levosulpiride was used in this test. 15 µM sertindole was included as a positive control. All groups were treated with agents for 48 h. Scale bar, 200 μm.

**Full-length blots**

**Figure 2**

**Figure 3**

**Figure 4**

**Figure 5**

**Supplementary Table S1** IC_50_ values of eight second-generation antipsychotic drugs toward SUM159 cells

| **NO.** | **Drugs** | **IC_50_ values (µM)** |
| --- | --- | --- |
| 1 | Asenapine | 55.3 |
| 2 | Blonanserin | >100.0 |
| 3 | Droperidol | >100.0 |
| 4 | Ziprasidone | >100.0 |
| 5 | Iloperidone | >100.0 |
| 6 | Amitriptyline | >100.0 |
| 7 | Sertindole | 9.2 |
| 8 | Clozapine | 62.8 |

**Supplementary Table S2 IC_50_ values of sertindole toward 30 cell lines**

| **Cancer type** | **Cell-Line names** | **IC_50_ values (µM)** |
| --- | --- | --- |
| **Lung cancer** | NCI-H460 | 6.2 |
|  | A549 | 11.2 |
|  | NCI-H446 | 15.2 |
|  | NCI-H661 | 12.2 |
|  | 801-D | 12.1 |
| **Glioblastoma** | U251 | 8.6 |
|  | A172 | 16.1 |
|  | U118-MG | 11.2 |
|  | U87-MG | 14.2 |
| **Gastric cancer** | AGS | 7.2 |
|  | MKN45 | 6.9 |
|  | BGC-823 | 10.3 |
|  | SGC-7901 | 14.2 |
| **Colon cancer** | HT-29 | 6.5 |
|  | COLO205 | 6.0 |
|  | SW480 | 15.1 |
|  | SW620 | 17.6 |
|  | HCT-15 | 9.7 |
| **Hepatoma** | HepG2 | 12.6 |
|  | Bel-7402 | 15.3 |
| **Breast cancer** | MCF-7 (ER^+^PR^+^HER2^-^) | 12.7 |
|  | MDA-MB-231 (ER^-^PR^-^HER2^-^) | 2.4 |
|  | SUM159 (ER^-^PR^-^HER2^-^) | 9.2 |
|  | T47D (ER^+^PR^+^HER2^-^) | 11.8 |
|  | MDA-MB-453 (ER^-^PR^-^HER2^+^) | 0.8 |
|  | ZR-75-1 (ER^+^PR^+^HER2^+^) | 3.4 |
| **Leukemia** | CCRF-CEM | 2.7 |
|  | K562 | 5.7 |
|  | Jurkat | 4.6 |
| **Immortalized breast**  **epithelial cells** | MCF-10A | 27.6 |
